# Identification of Five Serotypes of Enteropathogenic *Escherichia coli* from Diarrheic Calves and Healthy Cattle in Belgium and Comparative Genomics with Shigatoxigenic *E. coli*

**DOI:** 10.3390/vetsci9090492

**Published:** 2022-09-10

**Authors:** Audrey Habets, Fabrice Touzain, Pierrick Lucas, Nguyen Thi Thu Huong, Atsushi Iguchi, Florence Crombé, Nicolas Korsak, Denis Piérard, Marc Saulmont, Eric Cox, Frederik Engelen, Jacques Mainil, Damien Thiry

**Affiliations:** 1 Bacteriology, Department of Infectious Diseases, Faculty of Veterinary Medicine, Centre for Fundamental and Applied Research in Animals and Heath (FARAH), University of Liège, Quartier Vallée II, Cureghem Avenue 6, B-4000 Liège, Belgium; 2 Viral Genetics and Bio-Security Unit, French Agency for Food, Environmental and Occupational Health & Safety (ANSES), 22440 Ploufragan, France; 3 Department of Environment and Resource Sciences, University of Miyazaki (UoM), Miyazaki 889-2192, Japan; 4 Belgian National Reference Center STEC, Universitair Ziekenhuis Brussel (UZ Brussel), Vrije Universiteit Brussel (VUB), B-1090 Brussels, Belgium; 5 Food Microbiology, Department of Food Sciences, Faculty of Veterinary Medicine, Centre for Fundamental and Applied Research in Animals and Heath (FARAH), University of Liège (ULiège), B-4000 Liège, Belgium; 6 Regional Agency for Animal Health and Identification (ARSIA), B-5590 Ciney, Belgium; 7 Laboratory of Immunology, Department of Virology, Parasitology and Immunology, Faculty of Veterinary Medicine, Ghent University, Salisburylaan 133, B-9820 Merelbeke, Belgium

**Keywords:** *Escherichia coli*, EPEC, AE-STEC, diarrheic calves, healthy cattle, healthy calves

## Abstract

**Simple Summary:**

Enteropathogenic *Escherichia coli* (EPEC) from cattle receive little attention, although some belong to the most notorious O serotypes of attaching/effacing Shigatoxigenic *Escherichia coli* (AE-STEC) responsible for the uremic and hemolytic syndrome in humans, such as O26. Nevertheless, the O serotypes and virulotypes of the large majority of bovine EPEC remain unidentified. This study aimed to identify five non-classical O serotypes (O123/186, O156, O177, O182, and O183) by a polymerase chain reaction (PCR) among three collections of bovine EPEC from young diarrheic calves, healthy cattle at slaughterhouses, and healthy calves in dairy farms. The virulotypes and sequence types (STs) obtained after the whole genome sequencing of several O156, O177, and O182 bovine EPEC were closely related or identical to the virulotypes and STs of ten bovine and the human AE-STEC of the same O:H serotype. This study allows us to identify more EPEC O serotypes from cattle and to speculate on their evolution.

**Abstract:**

Enteropathogenic *Escherichia coli* (EPEC) produce attaching/effacing (AE) lesions and cause non-bloody diarrhea in mammals. A minority of bovine EPEC belong to one of the ten classical serotypes of human and bovine AE-STEC. The purpose of this study was to identify five non-classical O serotypes (O123/186, O156, O177, O182, and O183) among bovine EPEC and to characterize their virulence repertoires by whole genome sequencing. Around 40% of the 307 EPEC from 307 diarrheic calves, 368 EPEC from 47 healthy cattle, and 131 EPEC from 36 healthy calves in dairy farms were analyzed. Serotype O177 was the most frequent among EPEC from diarrheic and healthy calves, while the O156 was the most frequent in healthy cattle. The genomic analysis identified different H serotypes, MLSTypes, and/or *eae* gene subtypes among the O156 and O177 EPEC, while the O182 was homogeneous. The virulence gene profiles of bovine EPEC were closely related to each other and to the profiles of ten bovine and human AE-STEC. These results emphasize the need for additional studies to identify more O:H serotypes of bovine EPEC and to elucidate their origin and evolution of EPEC with regard to AE-STEC belonging to the same O:H serotypes.

## 1. Introduction

Enteropathogenic *Escherichia coli* (EPEC) are responsible for non-bloody diarrhea in humans and several animal species. They produce “Attaching/Effacing” (AE) histological lesions and harbor a pathogenicity island (the Locus of Enterocyte Effacement, or LEE), carrying genes coding for a type-3-secretion-system (T3SS), different type-3-secreted-effectors (T3SE), and the intimin adhesin (*eae*). EPEC are subdivided into “typical (t) EPEC,” producing the “Bundle Forming Pili” (BFP), type 4 fimbriae, and “atypical (a) EPEC” not producing the BFP. tEPEC are associated with humans, while aEPEC are isolated from humans and from different animal species, including young diarrheic calves and healthy cattle [1,2].

Enterohemorrhagic *Escherichia coli* (EHEC) are also important human pathogens responsible for hemorrhagic colitis (HC) and haemolytic uraemic syndrome (HUS). Since EHEC produce both AE lesions and Shiga toxins (Stx) and harbor the eae gene and one or more stx genes located on lambdoid phages (Stx phages) [2], they will be called “Attaching/Effacing Shigatoxigenic *E. coli*” (AE-STEC) in this manuscript [3]. The most frequent and pathogenic human AE-STEC belong to the “gang of seven” serotypes (O26:H11, O103:H2, O111:H-, O121:H9, O145:H-, O157:H7, and O165:H25), while the majority of AE-STEC isolated from diarrheic calves belong to a few serotypes (O5:H-, O26:H11, O103:H2, O111:H-, and O118:H16) [1,2,3,4].

EPEC can belong to host-specific (such as O127 in humans or O15 in rabbits) or to host-non-specific serotypes, including the “gang-of-seven” serotypes, such as O26:H11 and O111:H- in calves [1,2]. However, bovine EPEC have received little attention compared to bovine and human AE-STEC, and the serotypes of the majority of bovine EPEC remain unidentified. Following genetic serotyping results of a dozen of unidentified AE-STEC and EPEC from diarrheic calves [4], six new serotypes were identified: O80, O123/186, O156, O177, O182, and O183 (Iguchi and Mainil, unpublished). 

Studies on the O80 serotype have been published elsewhere [5]. The purpose of this study was, therefore, to (i) identify the other five new O serotypes amongst EPEC isolated from diarrheic calves, healthy calves, and healthy adult cattle; (ii) genetically compare these bovine EPEC with human and bovine AE-STEC belonging to the same serotypes.

## 2. Material and Methods

### 2.1. Previous Studies

The EPEC, from healthy cattle and calves, but not all from diarrheic calves, had already been tested with all PCRs for the “gang of seven” and three other O serotypes (O5, O80, and O118) [4,6,7], the ten most frequent and pathogenic STEC serogroups in humans and animals [8,9]. Therefore, a new list of priority serotypes of human and calf AE-STEC is proposed in this paper: the “gang of ten” (O5:H-, O26:H11, O80:H2, O103:H2, O111:H-, O118:H16, O121:H19, O145:H-, O157:H7, and O165:H25).

### 2.2. Escherichia coli Collections

EPEC, AE-STEC, and STEC were identified by a PCR for the *eae*, *stx1*, and *stx2* genes amongst 3 collections of *Escherichia coli* isolated from 307 diarrheic calves between 2009 and 2018 [4], from 368 healthy cattle at slaughterhouses between 2014 and 2015 [6], and from 131 healthy calves in dairy farms between June and October 2018 [10,11]. The strains from diarrheic calves were isolated from feces or intestinal contents of diarrheic and/or septicemic calves following the routine diagnostic procedure at ARSIA (Regional Agency for Animal Health and Identification), as reported elsewhere [4]. The other strains were isolated from intestinal samples of healthy cattle [6] and by the recto-anal mucosal swabbing of healthy calves [7].

### 2.3. Genomic O Serotyping

The first step was, therefore, to complete the PCR for the “gang of ten” O serotypes for the strains isolated from diarrheic calves. The “gang of ten” negative EPEC were subsequently tested with a PCR for the newly identified O serotypes (O123/186, which cannot be differentiated by the PCR), O156, O177, O182, and O183), according to the published O serotype PCR scheme [8].

All O serotyping PCRs were carried out in a 25 μL volume containing a 1 × FastGene Optima HotStart Ready Mix (Nippon Genetics Europe, Düren, Deutschland), primers (final concentration 10 μM, Eurogentec) (Table 1), and a 1 μL template DNA. The PCR was performed with the following amplification program: initial denaturation at 94 °C for 1 min followed by 35 cycles of denaturation at 94 °C for 30 s, then annealing at 58 °C for 30 s, extension at 72 °C for 1 min, and final extension at 72 °C for 2 min [7].

### 2.4. Whole Genome Sequencing

Genomic DNA was extracted (DNeasy^®^ Blood & Tissue kit, Qiagen, Venlo, Belgium) after overnight growth on Luria-Bertani (LB) agar plates from a single colony of different EPEC that tested positive for one of the newly identified O serotypes. The genomes of 6 bovine AE-STEC (5 from diarrheic calves and 1 from healthy cattle) and of 4 human AE-STEC (from the National Reference Center for STEC) belonging to the newly identified O serotypes were also sequenced for comparison.

Genomic libraries were prepared according to the Nextera XT kit (Illumina) and sequenced on an Illumina Novaseq 6000 (ANSES, Unité génétique virale et Biosécurité, Ploufragan, France) with an SP flowcell and a Reagent Kit v1.5 Kit (300 cycles). The length of the raw read was 151. The raw read sequences were assembled into scaffolds using the SPADes and Shovill genome assembler version 3.13.0 [12]. The genomic sequences are available via the Bioproject PRJNA607625 and the GenBank accession numbers (see Supplementary Table S1a–c)

The O:H serotype encoding genes and different LEE-located and non-LEE-located virulence-associated genes were identified in the sequenced isolates using two databases of the Centre of Genomic Epidemiology (CGE) tools (www.genomicepidemiology.org, accessed on 1 November 2021): SerotypeFinder 2.0 [13] and VirulenceFinder 2.0 [14]. The virulence genes were compared with Patric and Victors software [15]. The scaffolds of each isolate were tested with these tools with an identity threshold of 90% and a minimum length of 60%. The *eae* subtypes were detected with MyDb Finder 2.0 (see Supplementary Table S2) [16].

### 2.5. Phylogenetic Tree 

The seven housekeeping genes (*adk*, *fumC*, *icd*, *gyrB*, *mdh*, *purA*, and *recA*) present in the sequenced strains were identified using the MLST Server 2.0 database [17], also available via the Centre for Genomic Epidemiology (CGE) website. In order to study the phylogenetic relationship of the sequenced *E. coli* strains, the concatenated sequences of the 7 genes were aligned with the sequences of related reference genes from GenBank [4], maintaining a length of 3424 nucleotides using Muscle in MEGA11 software. The phylogenetic relationships of all sequences were analysed with MEGA11 software using the Maximum Likelihood method based on the Tamura–Nei model [18]. The statistical confidence for the tree was set by a bootstrap of 1000 replicates.

## 3. Results

### 3.1. Genomic O Serotyping

The “gang of ten” negative EPEC present in the three collections were subsequently tested by applying one triplex PCR for the O123/186, O156, and O177 serotypes and one duplex PCR for the O182 and O183 serotypes: 22% of EPEC isolated from the diarrheic calves, 22% of EPEC isolated from the healthy cattle, and 13% of EPEC isolated from the healthy calves tested positive. Serotypes O123/186, O177 (the most frequent), and O182 were detected amongst EPEC from the diarrheic calves and healthy calves compared to serotypes O156 (the most frequent), O177, and O182 amongst EPEC from the healthy cattle. No EPEC was positive with the PCR for the O183 serotype (see Supplementary Table S3a–c).

### 3.2. Identification of O:H Serotypes, MLSTypes, and Eae Gene Subtypes by WGS

Whole Genome Sequencing (WGS) was performed on thirty-four bovine EPEC and ten bovine or human AE-STEC for comparison. It included thirteen O156 (from the healthy cattle) along with two human and bovine AE-STEC, fifteen O177 (ten from the diarrheic calves, one from the healthy cattle, and four from the healthy calves) along with four human and bovine AE-STEC, six O182 (two from the diarrheic calves, two from the healthy cattle, and two from the healthy calves) along with four bovine and human AE-STEC.

Ten of the thirteen O156 EPEC, together with the two bovine and human AE-STEC, belonged to the O156:H25 serotype and carried the *eaeζ* gene subtype. However, the bovine EPEC and AE-STEC belonged to ST300 and the human AE-STEC to ST4942. The remaining three EPEC from the healthy cattle belonged to the O156:H8 serotype and ST327 and harbored the *eaeθ* gene subtype (see Supplementary Table S1a). 

Thirteen of the fifteen O177 EPEC belonged to the O177:H11 serotype and ST765, similarly to two bovine AE-STEC. The remaining two EPEC from the diarrheic calves belonged to the O177:H11 serotype and ST29 and to the O177:H25 serotype and ST342, like the human and the third bovine AE-STEC, respectively. Nevertheless, all 19 *eae* genes identified belonged to the *eaeβ* gene subtype (see Supplementary Table S1b).

All six O182 EPEC and four bovine and human AE-STEC belonged to the O182:H25 serotype and ST300 and carried the *eaeζ* gene subtype (see Supplementary Table S1c). 

### 3.3. Identification of Virulotypes by WGS

Using VirulenceFinder 2.0 with an identity threshold of 90% and a minimum length of 60%, different LEE-located T3SS- and T3SE-encoding genes, non-LEE-located T3SE-encoding genes, and genes coding for other virulence-associated factors were detected (Table 2).

Four LEE-located (*espA/espB/espF/tir/espP*) and four non-LEE-located genes (*nleA/B/C/espJ*) were detected in all or most of the EPEC. Conversely, the *cif*/*espI* genes were detected in all O177:H11/ST765 and in some O177:H11/ST29 and O177:H25/ST342 EPEC. The *cif* and *espI* genes were also detected in the three O156:H8/ST327 EPEC.

Similarly, different genes coding for other virulence-associated factors were detected in all of the EPEC, such as *gad*, *lpfA*, and *ehxA*, while other genes were associated with one or two serotypes, such as *etpD* with the O182:H25 and O156:H25 serotypes. Three genes (*katP*, *iha*, and *celb)* were detected in only a few of the EPEC (Table 2). The BFP type 4 fimbriae-encoding genes were not detected in any sequenced EPEC. The bovine and human AE-STEC had gene profiles identical or very similar to the gene profiles of the bovine EPEC belonging to the same serotype/ST, regardless of the *stx* subtype (Table 2).

### 3.4. Comparison of Serotypes, ST, and Virulotypes of EPEC from Diarrheic Calves, Healthy Cattle, and Healthy Calves

Differences between the three collections of EPEC were already present in the identified serotypes (ST1, 3). For instance, the serotypes O177:H11/H25 were more frequent in EPEC from calves that were either diarrheic or healthy than in healthy cattle, while the serotypes O156:H8/H25 were only present in EPEC from healthy cattle. 

**Figure 1:**
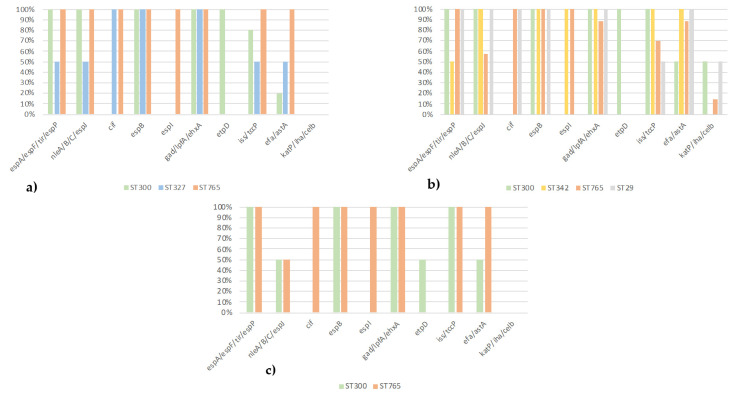
MLST prevalence by virulotyping in EPEC; (**a**) healthy cattle, (**b**) diarrheic calves, and (**c**) healthy calve strains.

Five STs (ST29, ST300, ST327, ST342, and ST765) were identified in the sequenced EPEC (Figure 1): four in EPEC from the diarrheic calves (ST29, ST300, ST342, and ST765), three in EPEC from the healthy cattle (ST300, ST327, and ST765) and two in EPEC from the healthy calves (ST300 and ST765). ST342 and ST29 were only present in the diarrheic calves, and ST327 was only present in the healthy cattle.

Concerning the virulotyping, the EPEC from the diarrheic calves harbored the most virulence genes (Figure 1). In the EPEC ST765 of serotype O177:H11 from the diarrheic calves, healthy cattle, and healthy calves, not only all of the LEE-located T3SS- and T3SE-encoding genes and different non-LEE-located T3SE-encoding genes were detected, but also different other genes coding for virulence-associated factors, except the *etpD* gene (Table 2). This *etpD* gene was detected only in EPEC ST300 of serotypes O156:H25 and O182:H25 in the three collections. The *efa1*/*astA* genes were preferentially associated with EPEC O177:H11/ST765 and O182:H25/ST300, while the other genes (*iss*/*tccP*/*katP*/*iha/celb*) were not associated with a serotype or STs or collection. 

Finally, the *eae* gene subtypes were associated with some STs (*eaeζ* and ST300, for instance) and to a lesser extent with the O:H (*eaeβ* and O177:H11/H25, for instance), but with none of the three collections (ST1).

### 3.5. Phylogenetic Analysis

A phylogenetic tree was constructed based on the seven housekeeping genes (*adk*, *fumC*, *icd*, *gyrB*, *mdh*, *purA*, and *recA*) of all the genome-sequenced O156, O177, and O182 bovine and human EPEC and AE-STEC. Strains of different serogroups, laboratory strains, or other bacterial strains were used to build the tree in order to know where the sequenced strains were located. 

With very few exceptions, all bovine and human EPEC and AE-STEC belonging to the same ST grouped together, while a second grouping basis was the H serotype. For instance, the EPEC and AE-STEC belonging to ST 29 (serotypes O177:H11), ST765 (serotype O177:H11), and ST327 (serotype O156:H8) were more closely related to each other than to those belonging to ST300 (serotypes O156:H25 and O182:H25). 

**Figure 2:**
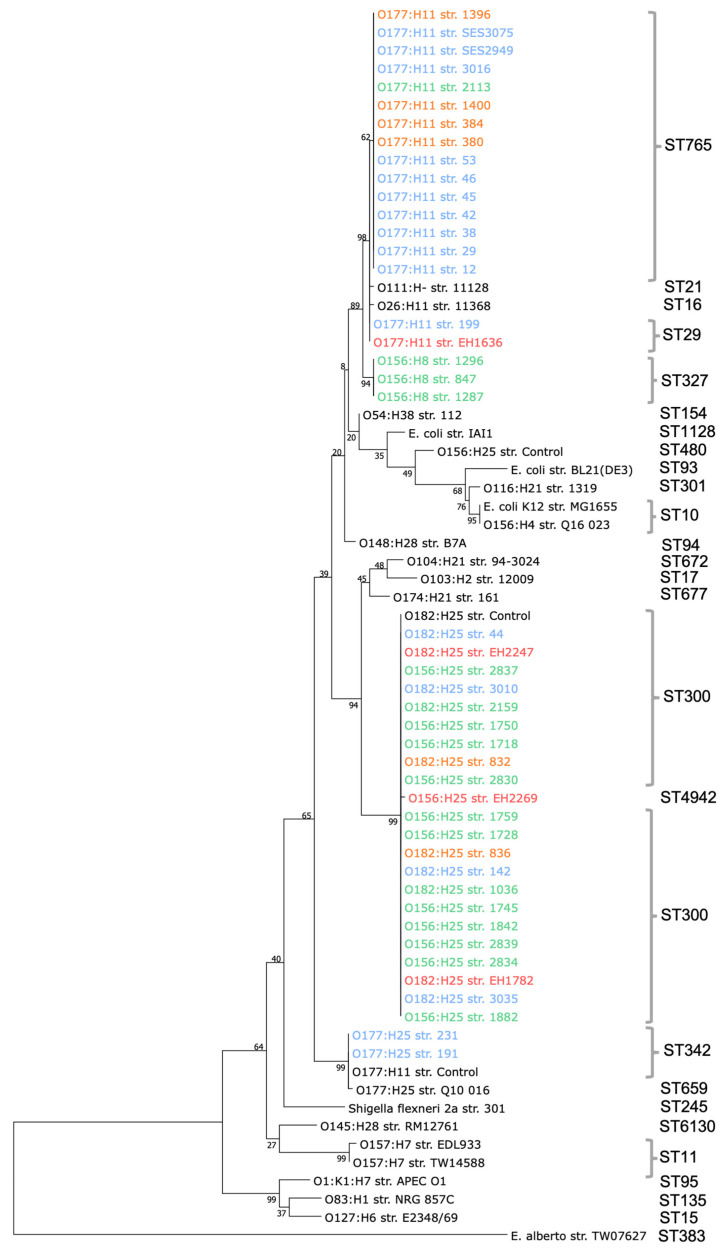
Phylogenetic tree of concatenated MLST gene alleles (*adk*, *fumC*, *icd*, *gyrB*, *mdh*, *purA*, and *recA*) extracted from the O156, O177. and O182. 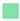 healthy cattle, 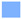 diarrheic calves, 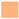 dairy calves, and 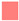 humans whose sequences of 3424 were compared to other strains based on [4,19]. The evolutionary history was inferred by using the Maximum Likelihood method based on the Tamura–Nei model and 1000 bootstrap replicates [18]. The percentage of trees in which the associated taxa clustered together is shown next to the branches.

Exceptions were (Figure 2): (i) the three bovine O156:H8/ST327 EPEC that were distantly related to the O156:H25/ST300 EPEC; (ii) the human AE-STEC O156:H25/ST4942 that grouped with all O182:H25/156:H25/ST300.

## 4. Discussion

If the large majority of bovine and human AE-STEC belong to one of the “gang of ten” O serotypes, this is not the case for the bovine EPEC, whose majority remain unidentified [1,2]. Indeed, only 31% of EPEC from the diarrheic calves, 11% from the healthy cattle at slaughterhouses, and 29% from the healthy calves in dairy farms are PCR positive for one of the “gang of ten” O serotypes in Belgium (ST3) ([4,6,7,16] and this study). The first purpose of this study was, therefore, to identify and compare, by PCR and WGS, additional serotypes (O123/186, O156, O177, O182, and O183) in these three collections of bovine EPEC.

Of the “gang of ten” negative EPEC, 11%, 23%. and 17%, respectively, belong to one of these serotypes (ST1, 3): serotypes O177:H11/H25 and O123/186 are (almost) exclusively associated with EPEC from calves, whether diarrheic or healthy, while serotypes O156:H8/H25 are exclusively present amongst EPEC from cattle at slaughterhouses. Conversely, serotype O182:H25 is present in EPEC of all three collections, and no EPEC tested positive for the O183 serotype. Some of these “non-classical” serotypes have already been detected, though infrequently, among human AE-STEC in Belgium [19] and abroad [20,21,22,23] in association with diseases. In 2006, an infection caused by an AE-STEC O177 was described in Canada in a child suffering from bloody diarrhea and HUS [21]. In 2018, in the USA, three AE-STEC isolated from diarrheic calves belonged to the O177:H25 serotype and harbored the *stx2* gene [22]. In Germany, the serotype O156:H25 is present among AE-STEC from humans with diarrhea [23]. 

From a clonality point of view, the STs are related to the serotypes of the EPEC, especially to the H antigen (Figure 2), and not to their origins. They are also present in bovine and/or human AE-STEC belonging to the same serotypes (ST1). The most frequent STs are ST300 and ST765, exclusively associated with serotypes O156:H25 and O182:H25 and with serotype O177:H11, respectively. In a recent study, ST300 and ST765 have also been detected in bovine AE-STEC strains belonging to serotypes O156:H25 and O182:H25 and to serotype O177:H11, respectively [24]. On the other hand, *E. coli* diarrheic strains, ST300, are more rarely found in humans [25]. In these same studies, ST342 and ST327 were identified among the bovine AE-STEC strains of serotypes O177:H25 and O156:H8, as were the four bovine EPEC and AE-STEC in this work [24], and ST29 among human EPEC [26], as is the case of one bovine EPEC and one human AE-STEC in this work (ST1).

The second purpose of this study was to compare the virulence-associated gene repertoires of the genome-sequenced EPEC (Table 2) of the three collections belonging to serotypes O156, O177, and O182. Three *eae* gene subtypes are present, but here, too, are associated with the O:H serotype and not with the collections. They are also present in bovine and/or human AE-STEC belonging to the same serotypes: *eaeθ* in the O156:H8 serotype, *eaeζ* in the O156:H25 and O182:H25 serotypes, and *eaeβ* in the O177:H11/H25 serotypes. It is noteworthy that the *eae* gene subtype of O156 EPEC is linked to the H serotype and ST, whereas this is not the case for O177 EPEC. 

Several LEE-located and non-LEE-located T3SS- and T3SE-encoding genes and different other virulence-associated genes are present in all 34 EPEC whose genomes were sequenced, some of which are ST-associated (Table 2a,b, Figure 1). Although the *espB* gene is present in all the strains, this gene was not detected with the Virulence Finder in 25 strains of the serotypes O156:H8, O156:H25, and O182:H25 (Table 2a). Since different *espB* variants can be present in EPEC and AE-STEC [26,27], we can hypothesize a lack of sensitivity of this software to detect all variants. Strains isolated from diarrheic calves have more potential virulence factors, such as *katP/iha/celB*, than those of the other two collections, suggesting that these genes would intervene at the level of the disease. The question that arises is: “is it a coincidence or did these strains emerge in these diarrheic calves as a result of a predisposing cause?”. The *iss* gene, which is a plasmid-located gene, a.o. on the pS88 plasmid of the invasive neonatal meningitis-associated *E. coli* (NMEC), and avian pathogenic *E. coli* (APEC) [28], is present in a large majority of the strains. However, the other pS88 plasmid-located genes were not detected, nor were other invasive-associated genes. The actual invasive capacity of these EPEC cannot be confirmed at this stage. Moreover, these 34 bovine EPEC are aEPEC since the BFP type 4 fimbriae-encoding genes were not detected. 

With regard to the *stx* genes, the *stx1a* gene is present in eight of the ten genome-sequenced bovine and human AE-STEC of different serotypes: the *stx2c* gene in one O177:H25 AE-STEC from a diarrheic calf and the *stx2g* in one bovine O156:H25 AE-STEC from the healthy cattle (ST1). The *stx2c* and *stx1a* genes are frequently detected in AE-STEC from cattle and humans, while the *stx2g* gene described in 2003 [29] is much rarer and is more frequently associated with cattle (healthy adults and diarrheic calves) and the environment than with humans [30,31,32]. 

Two questions need to be answered in the future: (i) Are the “non-gang of ten” EPEC really pathogenic in calves and/or in humans? (ii) Are they derivatives or precursors of AE-STEC? With regard to the former question, no or only a few data on these serotypes are available in the literature [16,21,22,23] to the authors’ knowledge, although some of them are clearly associated with diarrhea in calves in this study, such as O177:H11 (ST3a and 1b). As for the latter question, the 10 O156, O177, and O182 bovine and human AE-STEC, whose genomes were also sequenced, harbor identical to closely related virulence-associated gene repertoires compared to bovine EPEC belonging to the same serotypes (ST1 and Table 2). The presence of different *stx* genes in the 10 AE-STEC and the presence in a single animal of O156:H25 EPEC and AE-STEC (Table 2a) are also noteworthy. In the future, it would also be interesting to construct a phylogenetic tree based on the complete genomes, such as a single nucleotide polymorphism (SNP) [7], including even more sequences present in the Genbank. 

## 5. Conclusions

Of the five newly identified serogroups, O177 was the most frequent among EPEC from diarrheic and healthy calves, while O156 was the most frequent in healthy cattle. The genomic analysis identified different H serotypes, MLSTypes, and/or *eae* gene subtypes among the O156 and O177 EPEC, while the O182 was homogeneous. The virulence gene profiles of bovine EPEC were closely related to each other and to the profiles of 10 bovine and human AE-STEC.

Although this study was conducted on a limited number of strains, the results emphasize that bovine EPEC, not only of the AE-STEC “gang of ten” but also of other serotypes, deserve more attention in epidemiological surveys and in genetics studies to identify: (i) all EPEC serotypes in cattle; (ii) the origin of the infection in calves, the calves themselves, or the cows in the farms; (iii) their actual role in calf diarrhea; (iv) the transmission routes to humans, if any, in order to prevent their spread; and (v) their evolution and relationship to bovine and human AE-STEC belonging to the same O:H serotypes. Increasing the knowledge of these rarer not only EPEC but also AE-STEC serotypes can help to anticipate and/or rapidly react to their emergence in humans, such as the O104:H4 STEC in Germany in 2011 [33] and the O80:H2 AE-STEC in France and Switzerland [34] more or less recently.

## Figures and Tables

**Table 1:** Primers used for the different PCRs.

Gene	Primer	Sequence 5′-3′	Amplicon Size (bp)	Reference
O123/186	O123/186-F	TTTCAACAGGTTCGAATGCC	362	[8]
	O123/186-R	CCCACCAATACCACTGGAATA		
O156	O156-F	GGAAAATGGAACATTTAGCGG	236	[8]
	O156-R	TCGGAGTGCCAACCAAAATA		
O177	O177-F	CCGATACACCGGATGGATTAT	427	[8]
	O177-R	AAGCCAGTACCCAGAACAGGA		
O182	O182-F	CGGTGATGGTTCTATTCTTGG	502	[8]
	O182-R	TGCTTGCACCAACTGTGTTA		
O183	O183-F	CGTGGTAACCAATTTCGCAA	666	[8]
	O183-R	GGGAATAACGAACGGTTTACA		

**Table 2:** Comparison of the type 3 secretion system (T3SS)-, type 3-secreted effector (T3SE)-encoding genes and other virulence-associated-encoding genes identified by WGS in the bovine EPEC and bovine and human AE-STEC belonging to the O156, O177, and O182 O serotypes.

O:H Serotype (Nr. Isolates)	MLST (Nr. Isolates)	Host (Nr. Isolates)	Virulotype (Nr. Isolates)	LEE-Located T3SS- and T3SE-Encoding Genes: Nr. +Ve Isolates		Non-LEE-Encoded T3SE-Encoding Genes: Nr. +Ve Isolates			Other Virulence Genes: Nr. +Ve Isolates				
				*espA/espF/tir/espP*	*espB*	*nleA/B/C/espJ*	*cif*	*espI*	*gad/lpfA/ehxA*	*etpD*	*iss/tccP*	*efa1/astA*	*katP/iha/celB*
O156:H8 (3)	ST327 (3)	HC (3)	*eaeθ* (3) ^b^	0 to 3	3°	0 to 3	3	0	3	0	0 to 3	0–1	0
O156:H25 (12)	ST300 (11)	HC (11)	*eaeζ* (10) ^c,d^	10	10°	10	0	0	10	10	6	0	0
			*eaeζ*, *stx2g* (1) ^c^	1	1°	1	0	0	1	1	1	0	0
	ST4942 (1)	HU (1)	*eaeζ*, *stx1a* (1)	1	1°	1	0	0	1	1	1	0–1	0
O177:H11 (17)	ST765 (15)	DC (8)	*eaeβ* (8)	8	8	2 to 8	8	8	7	0	4 to 7	7	0–1
		HC (1)	*eaeβ* (1)	1	1	1	1	1	1	0	1	1	0
		HCS (6)	*eaeβ*, *stx1a* (1)	1	1	1	1	1	1	0	1	1	0
			*eaeβ, stx2c (1)*,						1	0	1	1	0
			*stx2c* (1)	1	1	1	1	1	4	0	4	4	0
	ST29 (2)	DC (1)	*eaeβ* (4) ^e^	4	4	2 to 4	4	4	1	0	0–1	1	0–1
		HU (1)	*eaeβ* (1)	1	1	1	1	0	1	0	1	1	0–1
			*eaeβ*, *stx1a* (1)	1	1	1	0	0					
O177:H25 (2)	ST342 (2)	DC (2)	*eaeβ* (1)	0–1	1	1	0	1	1	0	1	1	0
			*eaeβ*, *stx2c* (1)	1	1	1	0	0	1	0	1	0	0
0O182:H25 (10)	ST300 (10)	DC (4)	*eaeζ* (2)	2	2°	2	0	0	2	2	2	1	0
			*eaeζ*, *stx1a* (2)	2	2°	2	0	0	2	2	2	0–2	0–1
		HC (2)	*eaeζ* (2)	2	2°	2	0	0	2	2	2	2	0
		HCS (2)	*eaeζ* (2) ^f^	2	2°	0–2	0	0	2	1–2	2	1–2	0
		HU (2)	*eaeζ*, *stx1a* (2)	2	2°	1–2	0	0	2	2	0–2	0	0

DC = diarrheic calf; HC = healthy cattle; HU = human; HCS = healthy calves. ^b^ Two of these three EPEC were isolated from a single animal. ^c^ Three of these eleven EPEC and the AE-STEC were isolated from a single animal. ^d^ Four of these ten EPEC were isolated from another single animal. ^e^ These 4 EPEC were isolated from 2 different animals. ^f^ These 2 EPEC were isolated from 1 single animal. ° Only detected by Patric/Victors.

## Data Availability

The data were submitted to the NCBI GenBank under accession number PRJNA607625.

## Supplementary Materials

The following supporting information can be downloaded at: https://www.mdpi.com/article/10.3390/vetsci9090492/s1, Table S1a: Serotypes, MLST and *eae* gene subtypes of the O156 EPEC and AE-STEC from diarrheic calves, healthy cattle, healthy calves and humans; Table S1b: Serotypes, MLST and *eae* gene subtypes of the O177 EPEC and AE-STEC from diarrheic calves, healthy cattle, healthy calves and humans; Table S1c: Serotypes, MLST and *eae* gene subtypes of the O182 EPEC and AE-STEC from diarrheic calves, healthy cattle, healthy calves and humans; Table S2: Sequences included in the databases to identify *eae* subtypes; Table S3a: O serotypes of 126 EPEC isolated from calves with diarrhea; Table S3b: O serotypes of 122 EPEC isolated from healthy cattle at slaughterhouse; Table S3c: O serotypes of 60 EPEC isolated from healthy calves in dairy farms.
