# Supplementary material for: Identification of Five Serotypes of Enteropathogenic Escherichia coli from Diarrheic Calves and Healthy Cattle in Belgium and Comparative Genomics with Shigatoxigenic E. coli
Source: Vet Sci. 2022 Sep 10;9(9):492. doi: 10.3390/vetsci9090492 (PMC9505469; doi:10.3390/vetsci9090492)
Supplement: Supplementary file 1 [file vetsci-09-00492-s001.zip › vetsci-1819184-supplementary.pdf]

Table S1a. Serotypes, MLST and *eae* gene subtypes of the O156 EPEC and AE-STEC from diarrheic calves, healthy cattle, healthy calves and humans.

Isolate (year)	Host[a]	O:H serotype	MLST	*eae, stx* subtypes	GenBank accession numbers
847 (2014)	HC	O156:H8	ST327	*eaeθ*	SAMN14278591
1287 (2014)[b]	HC	O156:H8	ST327	*eaeθ*	SAMN14278592
1296 (2014)[b]	HC	O156:H8	ST327	*eaeθ*	SAMN14278593
1718 (2014)	HC	O156:H25	ST300	*eaeζ*	SAMN14278594
1728 (2014)[c]	HC	O156:H25	ST300	*eaeζ*	SAMN14278595
1745 (2014)[c]	HC	O156:H25	ST300	*eaeζ*	SAMN14278596
1759 (2014)[c]	HC	O156:H25	ST300	*eaeζ*	SAMN14278597
1842 (2014)	HC	O156:H25	ST300	*eaeζ*	SAMN14278598
1882 (2014)	HC	O156:H25	ST300	*eaeζ*	SAMN14278599
2830 (2014)[d]	HC	O156:H25	ST300	*eaeζ*	SAMN14278600
2834 (2014)[d]	HC	O156:H25	ST300	*eaeζ*	SAMN14278601
2837 (2014)[d]	HC	O156:H25	ST300	*eaeζ*	SAMN14278602
2839 (2014)[d]	HC	O156:H25	ST300	*eaeζ*	SAMN14278603
1750 (2014)[c]	HC	O156:H25	ST300	*eaeζ, stx2g*	SAMN14278604
EH2269 (2013)	HU	O156:H25	ST4942	*eaeζ, stx1a*	SAMN14278609

[a]DC = diarrheic calf; HC = healthy cattle; HU = human

[b]these 2 EPEC were isolated from a single animal

[c]these 3 EPEC and the AE-STEC were isolated from a single animal

[d]these 4 EPEC were isolated from a single animal

Table S1b. Serotypes, MLST and *eae* gene subtypes of the O177 EPEC and AE-STEC from diarrheic calves, healthy cattle, healthy calves and humans

Isolate (year)	Host[a]	O:H serotype	MLST	*eae, stx* subtypes	GenBank accession numbers
199 (2013)	DC	O177:H11	ST29	*eaeβ*	SAMN14278583
231 (2014)	DC	O177:H25	ST342	*eaeβ*	SAMN14278582
12 (2008)	DC	O177:H11	ST765	*eaeβ*	SAMN14278574
29 (2009)	DC	O177:H11	ST765	*eaeβ*	SAMN14278575
38 (2009)	DC	O177:H11	ST765	*eaeβ*	SAMN14278576
42 (2009)	DC	O177:H11	ST765	*eaeβ*	SAMN14278577
45 (2009)	DC	O177:H11	ST765	*eaeβ*	SAMN14278578
46 (2009)	DC	O177:H11	ST765	*eaeβ*	SAMN14278579
53 (2009)	DC	O177:H11	ST765	*eaeβ*	SAMN14278580
3016 (2015)	DC	O177:H11	ST765	*eaeβ*	SAMN14278583
2113 (2014)	HC	O177:H11	ST765	*eaeβ*	SAMN14278590
380 (2018)[b]	HCS	O177:H11	ST765	*eaeβ*	SAMN29328416
384 (2018)[b]	HCS	O177:H11	ST765	*eaeβ*	SAMN29328417
1396 (2018)[c]	HCS	O177:H11	ST765	*eaeβ*	SAMN29328418
1400 (2018)[c]	HCS	O177:H11	ST765	*eaeβ*	SAMN29328419
191 (2013)	DC	O177:H25	ST342	*eaeβ, stx2c*	SAMN14278584
SES2949 (2009)	DC	O177:H11	ST765	*eaeβ, stx1a*	SAMN29328420

SES3075 (2013)	DC	O177:H11	ST765	*eaeβ, stx1a*	SAMN29328421
EH1636 (2008)	HU	O177:H11	ST29	*eaeβ, stx1a*	SAMN14278608

[a]DC = diarrheic calf; HC = healthy cattle; HU = human; HCS: healthy calves
[b]these 2 EPEC were isolated from a single animal
[c]these 2 EPEC were isolated from a single animal

Table S1c. Serotypes, MLST and *eae* gene subtypes of the O182 EPEC and AE-STEC from diarrheic calves, healthy cattle, healthy calves and humans

Isolate (year)	Host[a]	O:H serotype	MLST	*eae, stx* subtypes	GenBank accession numbers
44 (2009)	DC	O182:H25	ST300	*eaeζ*	SAMN14278587
3035 (2014)	DC	O182:H25	ST300	*eaeζ*	SAMN14278587
1036 (2014)	HC	O182:H25	ST300	*eaeζ*	SAMN14278605
2159 (2014)	HC	O182:H25	ST300	*eaeζ*	SAMN14278606
832 (2018)[b]	HCS	O182:H25	ST300	*eaeζ*	SAMN29328414
836 (2018)[b]	HCS	O182:H25	ST300	*eaeζ*	SAMN29328415
142 (2012)	DC	O182:H25	ST300	*eaeζ, stx1a*	SAMN14278588
3010 (2014)	DC	O182:H25	ST300	*eaeζ, stx1a*	SAMN14278589
EH1782 (2009)	HU	O182:H25	ST300	*eaeζ, stx1a*	SAMN14278610
EH2247 (2013)	HU	O182:H25	ST300	*eaeζ, stx1a*	SAMN14278607

[a]DC = diarrheic calf; HC = healthy cattle; HU = human; HCS: healthy calves
[b]these 2 EPEC were isolated from a single animal

Table S2: Sequences included in the databases to identify *eae* subtypes

**Gene**	**Accession number**	**Description**
eae subtype α	AF022236.1	Intimin subtype, adhesin
eae subtype α	AF530555.1	Intimin subtype, adhesin
eae subtype β	AF453441.1	Intimin subtype, adhesin
eae subtype β	AF043226.1	Intimin subtype, adhesin
eae subtype ϒ	AF071034.1	Intimin subtype, adhesin
eae subtype ϒ/θ	AF025311.1	Intimin subtype, adhesin
eae subtype δ/κ	U66102.1	Intimin subtype, adhesin
eae subtype ε	AF116899.1	Intimin subtype, adhesin
eae subtype ζ	AF449417.1	Intimin subtype, adhesin
eae subtype η	AJ308550.1	Intimin subtype, adhesin
eae subtype ι	AJ308551.1	Intimin subtype, adhesin
eae subtype λ	AF530557.1	Intimin subtype, adhesin
eae subtype μ	AJ579305.1	Intimin subtype, adhesin
eae subtype ν	AJ579306.1	Intimin subtype, adhesin
eae subtype ξ	AJ705051.1	Intimin subtype, adhesin

Table S3a. O serotypes of 126 EPEC isolated from calves with diarrhea

O Serotype	Nr. Isolates EPEC (n=307) (%)	Reference
O5	0	4, 5, 16
O26	35 (11%)	This study
O80	54 (18%)	
O103	1 (<1%)	
O111	0	
O118	0	
O121	2 (<1%)	
O145	1 (<1%)	
O157	0	
O165	0	
O123/186	10 (3%)	This study
O156	0	
O177	21 (7%)	
O182	2 (<1%)	
O183	0	
Total	126 (41%)	

Table S3b. O serotypes of 122 EPEC isolated from healthy cattle at slaughterhouse

O Serotype	Nr. Isolates EPEC (n=368) (%)	Nr. Animals (= 47)	Reference
O5	0	0	7
O26	2 (<1%)	2	
O80	0	0	
O103	38 (12%)	7	
O111	0	0	
O118	0	0	
O121	0	0	
O145	0	0	
O157	0	0	
O165	0	0	
O123/186	0	0	This study
O156	67 (22%)	9	
O177	5 (2%)	1	
O182	10 (3%)	3	
O183	0	0	
Total	122 (40%)	22	

Table S3c. O serotypes of 60 EPEC isolated from healthy calves in dairy farms

O Serotype	Nr. Isolates (n=131) (%)	Nr. Animals (= 36)	Reference
O5	0	0	8
O26	4 (3%)	3	
O80	0	0	
O103	4 (3%)	2	
O111	5 (4%)	4	
O118	0	0	
O121	0	0	
O145	21 (16%)	9	
O157	4 (3%)	1	
O165	0	0	
O123/186	2 (2%)	1	This study
O156	0	0	
O177	17 (13%)	4	
O182	3 (2%)	1	
O183	0	0	
Total	60 (46%)	25	
